# Methods to assess helicase and translocation activities of human nuclear RNA exosome and RNA adaptor complexes

**DOI:** 10.1016/bs.mie.2022.03.060

**Published:** 2022-04-27

**Authors:** M. Rhyan Puno, Christopher D. Lima

**Affiliations:** 1 Structural Biology Program, Sloan Kettering Institute, Memorial Sloan Kettering Cancer Center, 1275 York Avenue, New York, NY 10065, USA; 2 Howard Hughes Medical Institute, 1275 York Avenue, New York, NY 10065

**Keywords:** RNA exosome, helicase, translocase, RNA-protein complex, DExH-box, SF2

## Abstract

The nuclear RNA exosome collaborates with the MTR4 helicase and RNA adaptor complexes to process, surveil, and degrade RNA. Here we outline methods to characterize RNA translocation and strand displacement by exosome-associated helicases and adaptor complexes using fluorescence-based strand displacement assays. The design and preparation of substrates suitable for analysis of helicase and decay activities of reconstituted MTR4–exosome complexes are described. To aid structural and biophysical studies, we present strategies for engineering substrates that can stall helicases during translocation, providing a means to capture snapshots of interactions and molecular steps involved in substrate translocation and delivery to the exosome.

## 1. Introduction

**Figure 1.**
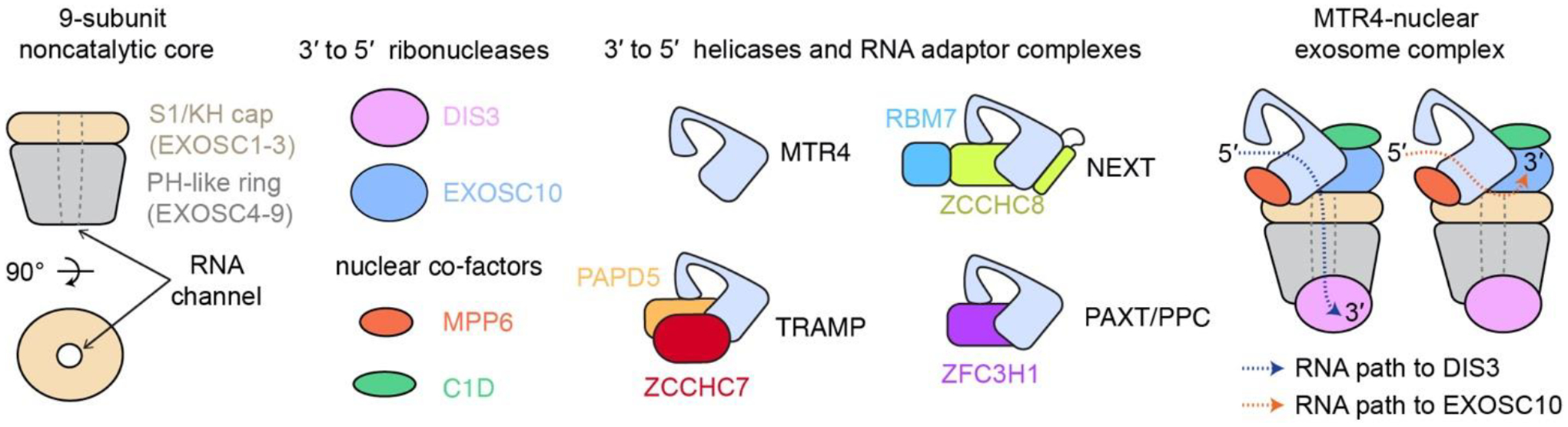
Human RNA exosome subunits, co-factors, and RNA adaptor complexes.

The RNA exosome is a conserved, eukaryotic 3′-to-5′ ribonuclease protein complex that is responsible for several aspects of nuclear and cytoplasmic RNA metabolism, ranging from general RNA turnover to processing and quality control of various coding and noncoding transcripts (Houseley, LaCava, & Tollervey, 2006; Puno, Weick, Das, & Lima, 2019). The human nuclear RNA exosome is composed of 9 core subunits with three S1/KH-domain proteins (EXOSC1–3) atop a hexameric ring of PH-domain proteins (EXOSC4–9) (Fig. 1). The exosome core contains a central channel that guides single-stranded RNA to two RNA-degrading enzymes: DIS3, a processive 3′-to-5′ RNAse II-like exoribonuclease and EXOSC10, a distributive 3′-to-5′ RNase D-like exoribonuclease that associate with the exosome core (Gerlach et al., 2018; Liu, Greimann, & Lima, 2006; Makino, Halbach, & Conti, 2013; Wasmuth, Januszyk, & Lima, 2014; Weick et al., 2018; Zinder & Lima, 2017).

Several functions of the nuclear RNA exosome require the helicase MTR4 (also known as SKIV2L2) (Weick & Lima, 2021). MTR4 is a multi-domain protein with a core structure composed of two RecA-like ATPase modules (RecA1 and RecA2), a winged helix (WH) fold and a C-terminal helical bundle (HB) domain (Gerlach et al., 2018; Jackson et al., 2010; Weick et al., 2018; Weir, Bonneau, Hentschel, & Conti, 2010). Inserted within WH is an arch-shaped protrusion consisting of an elongated stalk and a globular Kyprides-Ouzounis-Woese (KOW) domain that interacts with structured RNA and/or protein adaptors for exosome recruitment (Jackson et al., 2010; Taylor et al., 2014; Thoms et al., 2015). Nuclear cofactors MPP6 and/or C1D/EXOSC10 tether MTR4 to the exosome (Fig. 1) (Gerlach et al., 2018; Wasmuth, Zinder, Zattas, Das, & Lima, 2017; Weick et al., 2018).

MTR4 is a central component of several nuclear RNA adaptor complexes that identify, capture and prime exosome substrates for decay. In human, these include the Nuclear Exosome Targeting (NEXT) complex, the TRF4–2/PAPD5-ZCCHC7-MTR4 polyadenylation (TRAMP) complex, and the Poly(A) Exosome Targeting (PAXT) connection or Polysome Protector complex (PPC) (Fig. 1) (Lubas et al., 2011; Meola et al., 2016; Ogami et al., 2017). NEXT, composed of an RNA binding protein RBM7, a zinc-knuckle protein ZCCHC8, and the MTR4 helicase, functions in turnover of unwanted transcripts produced by pervasive transcription and surveillance of aberrant RNA species that emerge from faulty transcription. PAXT/PPC core is a binary complex between MTR4 and ZFC3H1 and is loosely associated other cofactors that include poly(A) binding protein PABPN1. PAXT/PPC primarily targets processed polyadenylated transcripts. In contrast to its *S. cerevisiae* counterpart, less is known about endogenous targets of human TRAMP.

The ability of MTR4 and RNA adaptor complexes to disrupt RNA structures and displace proteins deposited on RNA is key to their function in promoting exosome-mediated decay (Puno et al., 2019; Puno & Lima, 2018; Weick & Lima, 2021). In this chapter, we present methods used in our laboratory to characterize helicase activities of exosome-associated helicases and RNA adaptor complexes to determine mechanisms and kinetic models underpinning substrate selection, translocation, unwinding, and targeting to the exosome. These assays may also prove useful in studies aimed at assessing helicases and RNA adaptor complexes for their ability to displace proteins bound to RNA. We also provide a protocol for analyzing helicase-dependent RNA decay by MTR4–exosome complexes. Finally, we describe strategies in designing substrates for trapping helicases during translocation to generate samples for biophysical and structural analysis.

## 2. RNA helicase assays with MTR4 and RNA adaptor complexes

Assays that detect strand displacement are widely used to query translocation and/or helicase activity in vitro. These assays measure the ability of a helicase to displace and release an oligonucleotide strand (reporter strand) from a duplex region of a substrate. Unwinding polarity and requirements for a single-stranded translocation strand can be assessed by designing duplex substrates with single-stranded regions extending from either 5′ or 3′ ends.

**Figure 2.**
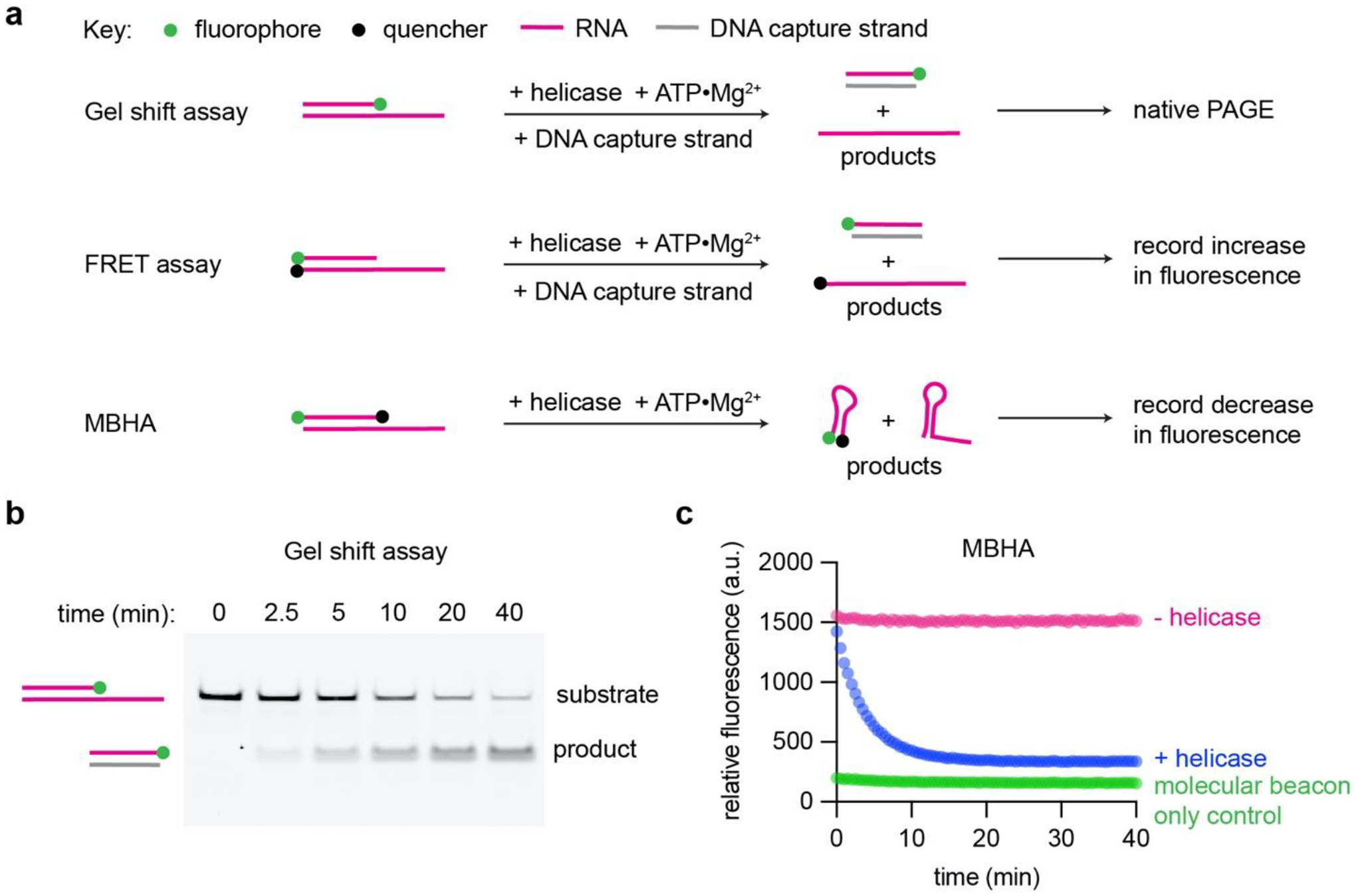
Strand displacement assays. (a) Schematics comparing gel shift, FRET, and MBHA assays. (b) Representative native PAGE showing time courses of helicase unwinding reaction on a 3′ tailed RNA duplex substrate. (c) MBHA fluorescence change in the presence or absence of helicase.

Several approaches are employed to monitor strand displacement using radiolabeled or fluorescently modified oligonucleotides. In a gel shift assay, the displaced reporter strand is captured by a complementary DNA oligonucleotide to prevent reannealing to the translocation strand while capturing the product for detection using native polyacrylamide gel electrophoresis (Fig. 2A and B). This is a common and accessible approach, since it utilizes materials and equipment available in most molecular biology laboratories, but it is limited by the number of samples and individual time points that can be conveniently taken manually. Throughput and temporal resolution are enhanced by using Fluorescence Resonance Energy Transfer (FRET)-based assays that enable detection of strand displacement in real time (Özeş, Feoktistova, Avanzino, Baldwin, & Fraser, 2014). Kinetic data can be obtained in minutes instead of hours as is often required for shift assays where assays are followed by gel electrophoresis and scanning. FRET substrates typically consist of a fluorescent reporter strand annealed to a translocation strand conjugated with a quencher dye (Fig. 2A). Upon strand displacement, the fluorophore is now distant from the quencher resulting in an increase in total reporter fluorescence.

A variant of the FRET-based method, the Molecular Beacon Helicase Assay (MBHA), employs a displacement strand that forms a stem loop after being released from the translocation strand. The displacement strand (molecular beacon) is modified with the fluorophore and quencher moieties at opposite ends (Belon & Frick, 2008; Özeş et al., 2014). Strand displacement results in a decrease in total fluorescence that can be detected in real time (Fig. 2A and C). The molecular beacon cannot reanneal to the translocation strand, so this approach effectively eliminates the need for a capture strand in the reaction. It also bypasses the synthesis of two modified oligonucleotides, reducing cost and expanding the size range of synthetic translocation strands that can be used.

The assays described in this section are performed under multiple turnover conditions whereby the helicase sequentially binds multiple substrates. For single turnover conditions, a helicase trap oligonucleotide is included in the reactions to prevent rebinding of dissociated helicase to the substrate (Pang, Jankowsky, Planet, & Pyle, 2002). Enzyme titrations are performed with concentrations generally ranging from 10-fold above and below the estimated RNA binding constant and can be used to calculate maximal rate and apparent substrate affinity (half maximal rate concentration).

### 2.1 Equipment

- C1000 Touch Thermal Cycler (Bio-Rad)
- Nanodrop 2000 (Thermo Fisher Scientific)
- SpectraMax M5 microplate reader (Molecular Devices)
- ThermoMixer (Eppendorf)
- Typhoon FLA 9500 scanner (GE Healthcare)
- XCell SureLock Mini-Cell tank (Thermo Fisher Scientific)

### 2.2 Materials

- Annealing buffer (10 mM Tris-HCl pH 7.0, 100 mM potassium acetate)
- 10X Assay buffer (100 mM Tris pH 7.0, 50% v/v glycerol, 15 mM MgCl_2_)
- ATP·MgCl_2_ solution (200 mM ATP, 200 mM MgCl_2_, pH adjusted to 7.0 using KOH and supplemented with in 1 mM Tris-HCl pH 7.0) (See Subsection 2.4 for details)
- Corning 3693 half-area 96-well white plate
- DNA capture strand (see Table 1, 8 μM Capture1 in annealing buffer)
- Lyophilized RNA (see Table 1; synthetic oligos obtained from IDT or Dharmacon)
- 2X Helicase solution (purified helicase in 20 mM Tris pH 7.0, 100 mM NaCl, 0.1 mM TCEP)
- Helicase diluent (20 mM Tris pH 7.0, 100 mM NaCl, 0.1 mM TCEP)
- Nuclease-free water
- 2X Quench solution (1% w/v sodium dodecyl sulfate, 10 mM ethylenediaminetetraacetic acid (EDTA), 10% v/v glycerol, 80 units/ml Proteinase K, 0.01% w/v xylene cyanol)
- Human placenta RNAse inhibitor (New England Biolabs)
- ß-mercaptoethanol (BME)
- 1X Tris-Borate-EDTA (TBE) running buffer (89 mM Tris-borate, 2 mM EDTA, pH 8.3)
- Novex 12-well 20% TBE polyacrylamide gel

### 2.3 Procedures

#### 2.3.1 Preparation of RNA substrates

1. Resuspend lyophilized oligonucleotides (GS1, GS2, MBHA1, and MBHA2 in Table 1) in annealing buffer to an estimated final concentration of 100 μM.
2. Incubate tube with resuspended RNA in ThermoMixer at 25 °C with a shake speed of 1000 rpm for 5 min. Transfer resuspended RNA into a fresh microcentrifuge tube.
3. Determine RNA concentration (in μM) by measuring absorbance at 260 nm (*A*_*260*_) using Nanodrop 2000. Calculate concentration using Eq. (1):
Eq. (1) $$A_{260}=\varepsilon bC$$
  where *ε* is the molecular extinction coefficient (M^−1^cm^−1^), *b* is the path length, and *C* is the concentration.
4. Prepare 8 μM of each RNA strand in annealing buffer.
5. Mix 25 μl of 8 μM reporter strand and 25 μl of 8 μM translocation strand in 0.2 ml PCR tube.
  - For the gel shift assay, mix oligonucleotide pairs GS1 and GS2 to generate the 3′ A_20_ tailed RNA duplex substrate. Prepare a 10X gel shift unwound control that will be used as a marker for captured reporter strand product: mix 19.5 μl annealing buffer, 0.5 μl of 8 μM GS1 and 20 μl 8 μM Capture1.
  - For the molecular beacon assay, mix oligonucleotide pairs MBHA1 and MBHA2 to generate the 3′ A_20_ tailed RNA duplex substrate. Prepare a 100X molecular beacon control: mix 35 μl annealing buffer and 5 μl of 8 μM MBHA1.
6. Using Bio-Rad C1000 Touch Thermal Cycler, heat the RNA mixture to 95 °C for 5 min followed by cooling to 16 °C for 10 minutes and 4 °C overnight. Transfer RNA mixture to a microcentrifuge tube and store at −80 °C until needed.
7. Analyze annealed RNA substrates using native polyacrylamide gel electrophoresis. Prepare 10 nM RNA substrate or reporter strand controls (GS1 or MBHA1) in 1X assay buffer. Mix 10 μl 10 nM RNA substrate or 10 nM reporter strand control with 10 μl of 2X quench solution. Load 10 μl sample into 12-well 20% Novex TBE polyacrylamide gel. Run gel in 1X TBE (pre-chilled to 4 °C) under constant voltage (200 V) for 1 hour at 4 °C. Scan gel for fluorescence using Typhoon FLA 9500 scanner. For 6-carboxyfluorescein (6-FAM) labeled substrates, scan the gel using FAM settings (473 nm laser, LPB filter). For cyanine 5 (Cy5), scan the gel using Cy5 settings (635 nm, LPR filter). More than 95% of the reporter strand should be annealed.

#### 2.3.2 Gel shift strand displacement assay

1. For each reaction, mix 13.5 μl nuclease-free water, 15 μl 10X assay buffer, 15 μl 50 mM BME and 1.5 μl 40 units/μl human placenta RNAse inhibitor.
2. Add 15 μl of 100 nM RNA solution to reaction tube. Mix well.
3. Prepare a 2X helicase solution in 20 mM Tris pH 7.0, 100 mM NaCl, 0.1 mM TCEP. Add 75 μl of 2X helicase solution in reaction mixture. Mix and incubate for 5 minutes at 22 °C. A protein titration is recommended with concentrations approximately 10-fold below and above the estimated equilibrium binding constant.
4. For the zero time point, pipet 18 μl reaction mixture into a microcentrifuge tube and add 2 μl 4 μM capture strand in annealing buffer and 20 μl quench solution.
5. Transfer 90 μl of the reaction mixture to a fresh microcentrifuge tube.
6. Prepare a fresh 10X ATP-capture solution. For 50 μl solution, mix 42.5 μl nuclease-free water, 5 μl 200 mM ATP·MgCl_2_ solution and 2.5 μl 8 μM DNA capture strand.
7. Initiate the reaction by adding 10 μl of 10X ATP-capture solution. Incubate the reaction tube in a ThermoMixer at 30 °C. Optional: Set shake speed to 30 rpm.
8. Take 18 μl aliquot of reaction mixture per time point and immediately transfer to a tube containing 18 μl 2X quench solution. Mix well.
9. Incubate quenched samples at 30 °C for 10 minutes to complete proteinase K digestion.
10. Resolve samples in Novex 12-well 20% TBE polyacrylamide gel. Rinse gel cassette with water. Place the gel cassette in XCell SureLock Mini-Cell tank and fill the upper and lower chambers with 1X TBE running buffer pre-chilled to 4 °C. Flush each well with 1X TBE running buffer. Load 10 μl sample into each well. For captured reporter strand marker, mix 13.5 μl nuclease-free water, 15 μl 10X assay buffer, 15 μl 50 mM BME and 1.5 μl 40 units/μl human placenta RNAse inhibitor, 15 μl 10X gel shift unwound control, 75 μl helicase diluent, 15 μl 20 mM ATP·MgCl_2_ solution. Load 10 μl captured reporter strand marker into a well. Run samples under constant voltage (200 V) for 1 hour at 4 °C.
11. Remove the gel from the cassette and scan for 6-FAM fluorescence (473 nm laser, LPB filter) using Typhoon FLA 9500 scanner. Save the scanned image as tif file.
12. Open tif file in ImageJ and quantify band intensities of unreacted substrate (*I*_*unreacted*_) and unwound product (*I*_*unwound*_). Subtract background fluorescence from each intensities.
13. Calculate fraction unwound (*f*) with background corrected (^bc^) intensities using the Eq. (2):
Eq. (2) $$f=\frac{I_{\text{unwound}}^{bc}}{I_{\text{unreacted}}^{bc}+I_{\text{unwound}}^{bc}}$$
14. Normalize data by subtracting fraction unwound at time = 0 due to nonenzymatic strand displacement from fraction unwound at each time point.
16. By plotting normalized fraction unwound (*f*_*norm*_) versus time (t), the data can be fitted to Eq. (3) (one-phase association equation in GraphPad Prism) to determine the observed unwinding rate constant *k*_*obs*_ and reaction amplitude (A):
Eq. (3) $$f_{\text{norm}}=A\left[1-\exp\left(-k_{obs}\cdot t\right)\right]$$
15. Initial unwinding rate can be calculated by taking the product of observed rate constant and reaction amplitude or by the taking the slope of fitted linear curve of data at early time points.

#### 2.3.3 Molecular beacon helicase assay

1. Prepare MBHA master mix: 162 μl nuclease-free water, 180 μl 10X assay buffer, 180 μl 50 mM BME, 18 μl human placenta RNAse inhibitor, 180 μl 100 nM RNA substrate (3′ A_20_ tailed MBHA substrate). This master mix is sufficient for 8 reactions with 3 technical replicates and can be used to assay unwinding at various helicase concentrations.
2. Pipet 20 μl MBHA master mix per reaction well into Corning 3693 half-area 96-well white plate. Use a multichannel pipette when conducting multiple reactions.
3. Prepare a 2X helicase solution in 20 mM Tris pH 7.0, 100 mM NaCl, 0.1 mM TCEP. Add 25 μl 2X helicase solution or helicase diluent (no protein control) into each reaction well. Mix by pipetting.
4. Prepare molecular beacon control solution: 69 μl nuclease-free water, 10 μl 10X assay buffer, 10 μl 50 mM BME, 1 μl human placenta RNAse inhibitor, 10 μl 100X molecular beacon control. Add 45 μl molecular beacon control solution into 3 replicate wells.
5. Insert plate into SpectraMax M5 microplate reader pre-heated to 30 °C and record fluorescence (excitation/emission wavelength = 643 nm/667 nm, medium photomultiplier tube (PMT) setting, 6 flashes per read) every 30 s for 5–10 min or until the fluorescence stabilizes.
6. Initiate the reaction by adding 5 μl 20 mM ATP·MgCl_2_ solution into each well using a multichannel pipette.
7. Record fluorescence every 30 s for 40 min at 30 °C.
8. Calculate normalized fraction unwound for each time point using Eq (4):
Eq. (4) $$f_{\text{norm}}=\frac{S_{t}-S_{0}}{S_{0}-M_{t}}-\frac{C_{t}-C_{0}}{C_{0}-M_{t}}$$
  where *S*_*t*_ is the sample fluorescence at time *t*, *S*_*0*_ is the sample fluorescence at time 0, *M*_*t*_ is the fluorescence of the molecular beacon control at time *t*, *C*_*t*_ is the fluorescence of no helicase control at time *t* and *C*_*0*_ is the fluorescence of no protein/helicase control at time 0.
9. Data are fitted to Eq. (3) using GraphPad Prism to determine the observed unwinding rate constant *k*_*obs*_ and the extent of the reaction (A).
10. Initial rate can be calculated by taking the product of the observed rate constant and reaction amplitude or by the taking the slope of fitted linear curve of data at early time points.

### 2.4 Notes

- Buffer conditions (buffer, pH, ionic strength) should be optimized for every helicase assayed.
- ATP·MgCl_2_ stock solution (200 mM) is prepared using ATP disodium salt hydrate lyophilized powder (Sigma). Hydrate content should be checked in the accompanying analysis certificate. Adjust the pH using potassium hydroxide and supplement with 1 mM Tris-HCl pH 7.0. Add stoichiometric amount of MgCl_2_ prior to storage at −80 °C.
- Fluorescence intensity of specific dyes can be reduced when placed adjacent to a guanine nucleotide.
- The influence of the position (5′ vs 3′ end of the reporter strand) of the fluorophore on helicase activity should be tested.
- Strand displacement assays are typically accompanied with measurements of nucleotide triphosphate (NTP) consumption to determine how energy expenditure is coupled to translocation. For some helicases, NTP hydrolysis is dispensable for strand displacement but is required for enzyme recycling (Liu et al., 2008). The number of NTP used per nucleotide translocation also vary among helicases. Several methods (Brune et al., 1994, Bradley and De La Cruz, 2012, Rajagopal and Lorsch, 2013, Özeş et al., 2014) and commercial kits are available to monitor NTPase activity.

## 3. RNA unwinding and decay assays of MTR4-exosome complexes

**Figure 3.**
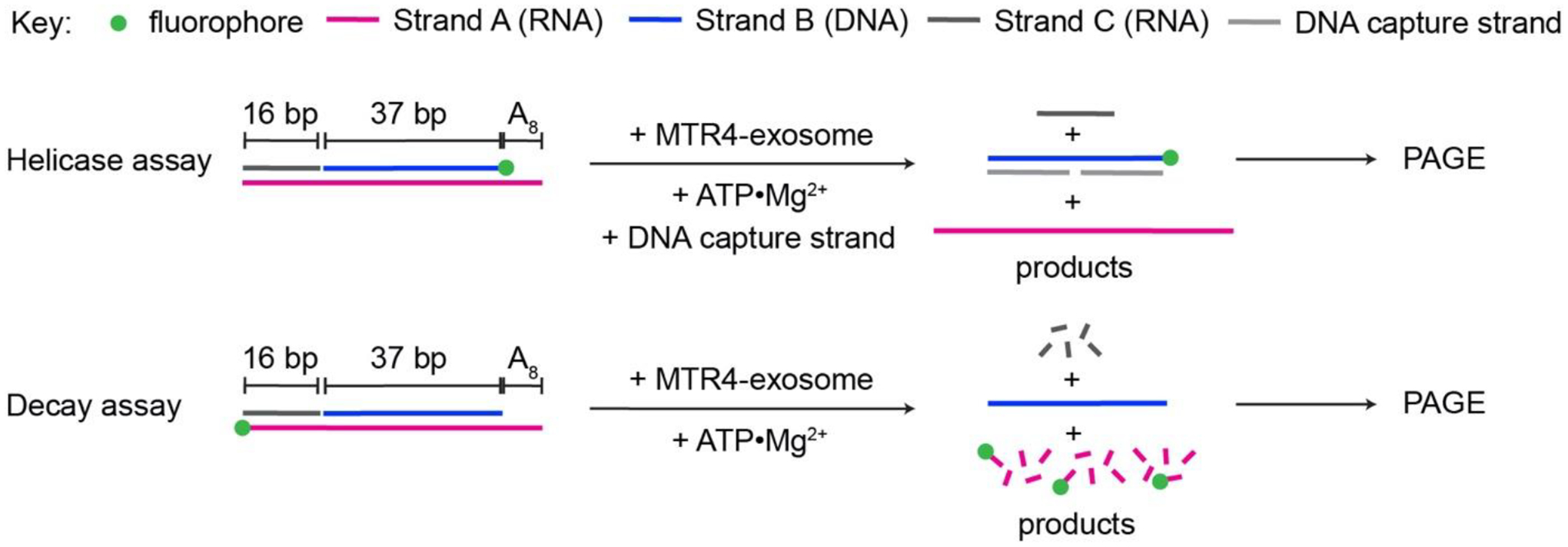
Substrate design and reaction scheme for RNA helicase and decay assay of human MTR4-exosome complex.

RNA unwinding by MTR4 and RNA adaptor complexes ultimately leads to 3′ end trimming or decay by the exosome. These processes are coupled through physical interaction of MTR4 with the exosome via nuclear co-factors MPP6 and/or the C1D/EXOSC10 heterodimer. To analyze RNA unwinding and decay activities of recombinant human MTR4-exosome complexes, we designed fluorophore-labeled tripartite substrates (Fig. 3, Table 1) that can be used for gel-based assays (Weick et al., 2018). For decay assays, the translocation strand (strand A) is labeled with a fluorescent moiety whereas for helicase assays, a DNA oligonucleotide (strand B) annealed to the translocation strand is conjugated with a fluorophore (Fig. 3). A short poly(A)_8_ overhang is included for interaction with MTR4 (Jia et al., 2011). These substrates can serve as templates for exploring conditions or modifications that alter MTR4-exosome activities. The following procedures describe substrate preparation and protocols for helicase and decay assays of MTR4-exosome complexes.

### 3.1 Equipment

- C1000 Touch Thermal Cycler (Bio-Rad)
- Nanodrop 2000 (Thermo Fisher Scientific)
- ThermoMixer (Eppendorf)
- Typhoon FLA 9500 scanner (GE Healthcare)
- Xcell SureLock Mini-Cell tank (Thermo Fisher Scientific)

### 3.2 Materials

- Annealing buffer (10 mM Tris pH 7.0, 100 mM potassium acetate)
- ATP·MgCl_2_ solution (200 mM ATP, 200 mM MgCl_2_, pH adjusted to 7.0 using KOH and supplemented with in 1 mM Tris-HCl pH 7.0) (See Subsection 2.4 for details)
- 50 mM ß-mercaptoethanol (BME)
- DNA capture strand (see Table 1, 3 μM Capture2 in annealing buffer)
- Lyophilized RNA (See Table 1; synthetic oligos from IDT or Dharmacon)
- Assay buffer (20 mM HEPES-KOH pH 7.5, 50 mM potassium acetate, 0.5 mM magnesium acetate, 2.5 mM DTT, 0.01% IGEPAL CA-630)
- Human placenta RNAse inhibitor (40 U/μl, New England Biolabs)
- Human DIS3/EXOSC10/MTR4-exosome protein complexes in assay buffer engineered with or without mutations in the DIS3 and/or EXOSC10 active sites that disrupt hydrolysis and catalytic activity for helicase or decay assays, respectively (Weick et al., 2018)
- 2X Quench solution (1% w/v sodium dodecyl sulfate, 10 mM ethylenediaminetetraacetic acid (EDTA), 10% v/v glycerol, 80 units/ml Proteinase K, 0.01% w/v xylene cyanol)
- 0.5X TBE running buffer (44.5 mM Tris-borate, 1 mM EDTA, pH 8.3)
- Novex 12-well 4–20% TBE polyacrylamide gel (Thermo Fisher Scientific)

### 3.3 Protocols

#### 3.3.1 Preparation of RNA substrates

1. Resuspend and prepare oligonucleotides (StrandA1, StrandA2, StrandB1, StrandB2, and StrandC1) as in steps 1–3 of Subheading 2.3.1.
2. Prepare a stock solution of 32 μM for each oligonucleotide.
3. For helicase substrate, mix 10 μl of 32 μM StrandA1, 10 μl of 32 μM StrandB2, 10 μl of 32 μM StrandC1 and 10 μl annealing buffer in 0.2 ml PCR tube. For decay substrate, mix 10 μl of 32 μM StrandA2, 10 μl of 32 μM StrandB1, 10 μl of 32 μM StrandC1 and 10 μl annealing buffer in 0.2 ml PCR tube. Prepare a 10X gel shift unwound control that will be used as a marker for captured reporter strand product: mix 35.5 μl annealing buffer, 0.5 μl of 8 μM StrandB2 and 4 μl 3 μM Capture2.
4. Using Bio-Rad C1000 Touch Thermal Cycler, heat the RNA mixture to 95 °C for 5 min followed by cooling to 16 °C for 10 minutes and 4 °C overnight. Transfer RNA mixture to a microcentrifuge tube and store at −80 °C until needed.
5. Analyze annealed RNA substrate using native polyacrylamide gel electrophoresis. Prepare 10 nM RNA substrate or fluorescent reporter strand control (StrandA2 or StrandB2) in 1X assay buffer. Mix 10 μl 10 nM RNA substrate or 10 nM reporter strand control with 10 μl of 2X quench solution. Load 10 μl sample into 12-well 20% Novex TBE polyacrylamide gel. Run gel in 1X TBE (pre-chilled to 4°C) under constant voltage (200 V) for 1 hour at 4 °C. Scan gel for 6-FAM/fluorescein fluorescence (473 nm laser, LPB filter). More than 95% of the fluorescent strand should be annealed.

#### 3.3.2 RNA unwinding assay of MTR4-exosome complexes

1. For each reaction, mix 69 μl assay buffer, 1 μl 40 units/μl human placenta RNAse inhibitor, 10 μl 100 nM RNA and 10 μl 200 nM MTR4-exosome complexes with catalytically inert ribonucleases (Weick et al., 2018) in a microcentrifuge tube.
2. Incubate at 20 °C for 5 min.
3. For sample at 0 time point, take 18 μl reaction mixture and mix with 2 μl assay buffer and 10 μl quench solution.
4. Transfer 54 μl reaction mixture to a fresh microcentrifuge tube.
5. Prepare a fresh 10X ATP-capture solution. For 50 μl solution, mix 42.5 μl nuclease-free water, 2.5 μl 200 mM ATP·MgCl_2_ solution and 5 μl 3 μM DNA capture strand.
6. Initiate the reaction by adding 6 μl ATP-capture solution.
7. Take 10 μl aliquot of reaction mixture at various time points and terminate the reaction by adding 5 μl quench solution.
8. Incubate quenched samples at 30 °C overnight to complete proteinase K digestion.
9. Rinse each well of a pre-cast Novex 12-well 4–20% TBE polyacrylamide gel with water.
10. Place the gel cassette in XCell SureLock Mini-Cell tank and fill the upper and lower chambers with 0.5X TBE running buffer (pre-chilled to 4 °C). Flush each well with 0.5X TBE running buffer.
11. Load 3.5 μl sample into each lane. For captured reporter strand marker, mix 39.5 μl assay buffer, 0.5 μl 40 units/μl human placenta RNAse inhibitor, 5 μl 10X gel shift unwound control, 5 μl 20 mM ATP·MgCl_2_, 50 μl quench solution. Load 3.5 μl captured reporter strand marker into a well.
12. Run samples under constant voltage (200 V) for 45 min at 4 °C.
13. Remove the gel from the cassette and scan for 6-FAM fluorescence (473 nm laser, LPB filter) using Typhoon FLA 9500 scanner.

#### 3.3.3 RNA decay assays of MTR4-exosome complexes

1. For each reaction, mix 69 μl assay buffer, 1 μl 40 units/μl human placenta RNAse inhibitor, 10 μl 100 nM RNA and 10 μl 20 mM ATP·MgCl_2_ of MTR4-exosome complexes in a microcentrifuge tube with or without mutations in EXOSC10 that disrupt its activity to facilitate analysis of DIS3 activities (Weick et al., 2018).
2. Incubate at 20 °C for 5 min.
3. For sample at 0 time point, take 18 μl aliquot of reaction mixture and mix with 2 μl assay buffer and 10 μl quench solution.
4. Pipet 54 μl reaction mixture to a fresh microcentrifuge tube.
5. Initiate the reaction by adding 6 μl 1 μM MTR4-exosome complex.
6. Take 10 μl aliquot of reaction mixture at various time points and stop the reaction by adding 5 μl quench solution.
7. Incubate quenched samples at 30 °C overnight to complete proteinase K digestion.
8. Rinse each well of a pre-cast Novex 12-well 4–20% TBE polyacrylamide gel with water.
9. Place the gel cassette in XCell SureLock Mini-Cell tank and fill the upper and lower chambers with 0.5X TBE running buffer. Flush each well with 0.5X TBE running buffer.
10. Load 3.5 μl sample into each lane.
11. Run samples under constant voltage (200 V) for 45 min at 22 °C.
12. Remove the gel from the cassette and scan for fluorescein fluorescence (473 nm laser, LPB filter)using Typhoon FLA 9500 scanner.

### 3.4 Notes

- Assay buffer can be replaced with 20 mM Tris-HCl pH 7.0, 50 mM NaCl, 0.5 mM MgCl_2_, 2.5 mM DTT, 0.01% IGEPAL CA-630 to avoid precipitation of SDS by potassium salt.

## 4. Preparation of RNA-loaded human MTR4-exosome complex for structural studies

**Figure 4.**
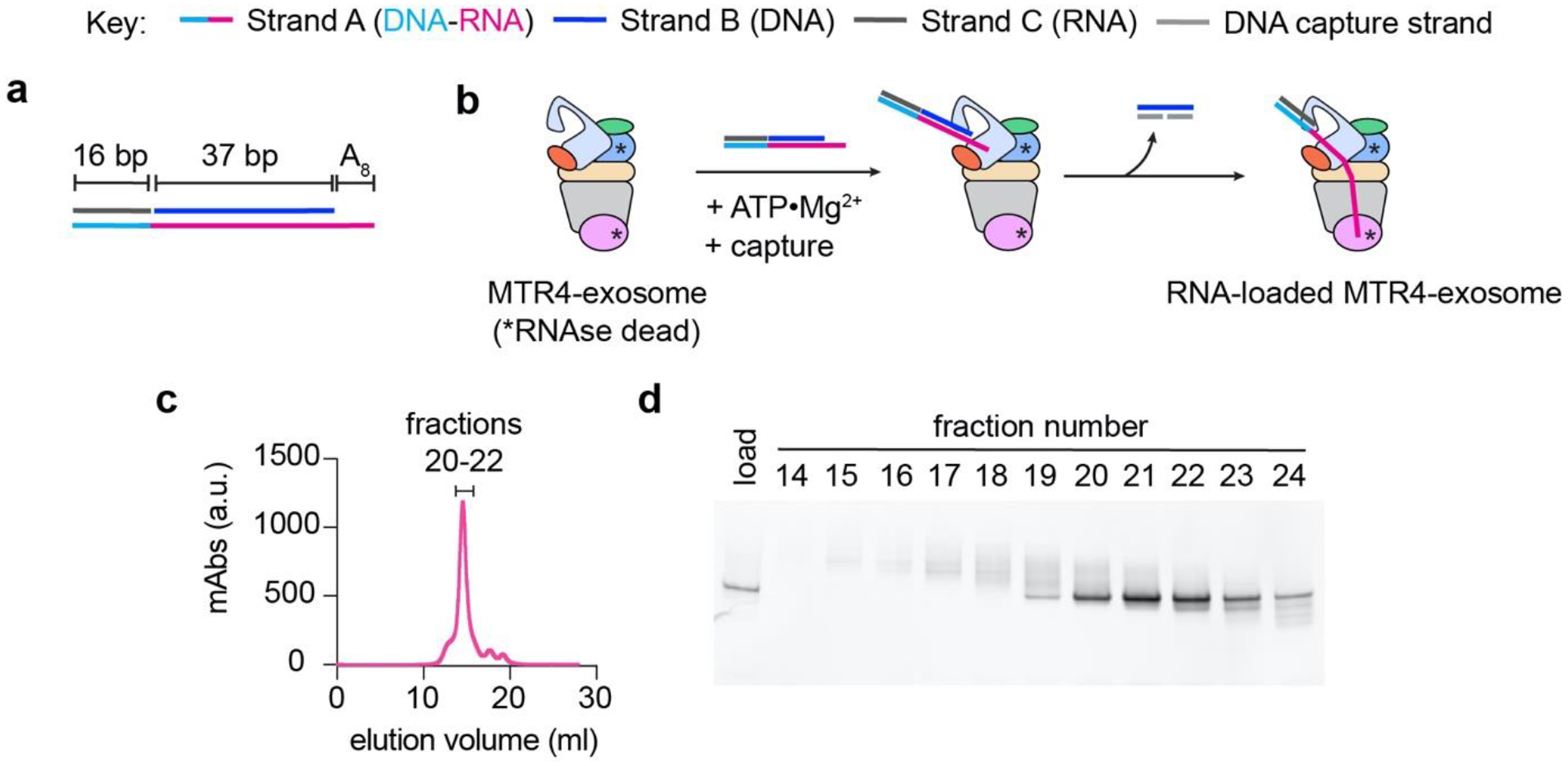
RNA loading to human MTR4-exosome complex. (a) DNA-RNA chimera tripartite substrate (b) Schematics showing the RNA loading strategy for human MTR4-exosome complex using the DNA-RNA chimera substrate. (c) Superdex S200 Increase 10/300 GL chromatogram of annealed DNA-RNA chimera tripartite substrate. (d) SYBR Gold-stained native PAGE showing Superdex S200 Increase fractions of purified DNA-RNA chimera tripartite substrate.

To understand how MTR4 and other exosome-associated helicases unwind and feed RNA into the exosome, we engineered substrates to capture the helicase during the course of RNA translocation. We previously reported use of a substrate with a chimeric DNA-RNA translocation strand (Fig. 4a) to determine the cryogenic electron microscopy (cryo-EM) structure of RNA-loaded human MTR4-exosome complex in the process of unwinding and delivering RNA to the exosome (Weick et al., 2018). The DNA-RNA translocation strand allows MTR4 tracking on the RNA segment of the substrate but induces stalling as the helicase reaches the DNA-RNA junction (Fig. 4b). We designed the length of the translocation strand based on the number of nucleotides accommodated by MTR4, exosome core, and DIS3 in reported crystal structures of *S. cerevisiae* homologs (Weir et al., 2010, Zinder et al., 2016). The optimal length was refined by performing a helicase reaction followed by a crosslinking assay (Weick et al., 2018). In this section, we describe the preparation and loading of a tripartite DNA-RNA chimera substate to MTR4-exosome complex for cryo-EM analysis.

### 4.1 Equipment

- ÄKTA pure 25 L chromatography system (GE Healthcare)
- Bio-Rad ChemiDoc XRS+
- C1000 Touch Thermal Cycler (Bio-Rad)
- Nanodrop 2000 (Thermo Fisher Scientific)
- ThermoMixer (Eppendorf)
- Typhoon FLA 9500 scanner (GE Healthcare)
- XCell SureLock Mini-Cell tank (Thermo Fisher Scientific)

### 4.2 Materials

- Lyophilized RNA (see Table 1; synthetic oligos from IDT or Dharmacon)
- Annealing buffer (10 mM Tris-HCl pH 7.0, 100 mM potassium acetate)
- ATP·MgCl_2_ (200 mM ATP, 200 mM MgCl_2_, pH adjusted to 7.0 using KOH and supplemented with in 1 mM Tris-HCl pH 7.0) (See Subsection 2.4 for details)
- DNA capture strand (see Table 1, 90 μM Capture2 in annealing buffer)
- ß-mercaptoethanol (BME)
- Human placenta RNAse inhibitor (New England Biolabs)
- Human DIS3/EXOSC10/MTR4-exosome complex with mutations that disrupt DIS3 and EXOSC10 exoribonuclease activity (Weick et. al., 2018; protein complex in 20 mM Tris-HCl pH 8.0, 50 mM NaCl, 0.5 mM MgCl2, 2.5 mM BME)
- 4X Lithium dodecyl sulfate (LDS) sample buffer (Thermo Fisher Scientific)
- Loading Buffer (20 mM Tris-HCl pH 8.0, 100 mM NaCl, 2.5 mM BME, 0.5 mM MgCl_2_, 20% v/v glycerol, 10 mM EDTA, 0.2% v/v SDS, 50 units/ml Proteinase K)
- Lyophilized oligonucleotides (see Table 1, synthetic oligos from IDT or Dharmacon)
- MES running buffer (Thermo Fisher Scientific)
- Novex 12-well 4–20% TBE polyacrylamide gel (Thermo Fisher Scientific)
- NuPage 4–12% Bis-Tris polyacrylamide gel (Thermo Fisher Scientific)
- 2X Quench solution (20 mM Tris-HCl pH 8.0, 50 mM NaCl, 6 mM AMPPNP, 1 mM BME)
- Superdex S200 Increase 10/300 GL (Thermo Fisher Scientific)
- SYBR Gold Gel Stain (Thermo Fisher Scientific)
- 1X TBE running buffer (89 mM Tris-borate, 2 mM EDTA, pH 8.3)

### 4.3 Protocol

#### 4.3.1 Substrate preparation

1. Resuspend lyophilized, HPLC-purified oligonucleotides in annealing buffer (10 mM Tris pH 7.0, 100 mM potassium acetate) to an estimated final concentration of 400 μM.
2. Follow steps 2–3 in Section 3.3.1.
3. Prepare 200 μl mixture of 100 μM StrandA3, 200 μM StrandB3, and 100 μM StrandC1. Aliquot 50 μl into a PCR 8-tube strip.
4. Using a thermocycler, incubate the RNA mixture at 95 °C for 5 min, 16 °C for 10 minutes then 4 °C overnight. Pool annealed RNA soluton into a 1.7 ml microcentrifuge tube.
6. Purify annealed RNA using Superdex S200 Increase 10/300 GL pre-equilibrated with annealing buffer. Representative chromatogram is shown in Fig. 4c.
7. Analyze fractions for nucleic acid content using native PAGE. Take 1 μl aliquot and add 19 μl of 1X TBE sample buffer. Resolve samples (10 μl/lane) in Novex 4–20% TBE polyacrylamide gel using pre-chilled 1X TBE running buffer (200 V for 1 hour at 4 °C). Stain gel with 1X SYBR Gold dissolved in 1X TBE for 10 minutes. Wash gel with 1X TBE for 10 minutes. Scan gel for fluorescence using Bio-Rad ChemiDoc XRS+. Representative native PAGE analysis of nucleic acids in Superdex S200 Increase fractions is shown in Fig. 4d.
8. Take peak fractions, determine RNA concentration, and store at −80 °C until needed.

#### 4.3.2 Preparation of RNA-loaded human MTR4-exosome complex

1. Mix 50 μl 9 μM human MTR4-exosome complex with 50 μl 9 μM substrate.
2. Prepare fresh ATP-capture solution. For 50 μl solution, mix 20 μl nuclease-fre water, 5 μl 200 μM 200 mM ATP·MgCl_2_ solution and 25 μl 90 μM DNA capture strand. Add 10 μl of 20 mM ATP-capture mix to initiate reaction.
3. Incubate reaction mixture at 22 °C for 2 h.
4. Add 50 μl of quench solution.
5. Spin at 14,000 × g using a microcentrifuge for 5 min at 4 °C.
6. Purify using Superdex 200 Increase 10/300 GL pre-equilibrated with SEC buffer.
7. Analyze fractions for protein content using SDS-PAGE. Take 15 μl aliquot and add 5 μl 4X LDS sample buffer supplemented with 5% v/v BME (710 mM BME). Heat at 95 °C for 3 min. Resolve samples in 4–12% NuPage Bis-Tris gel using 1X MES running buffer. Stain gel with Coomassie staining solution. Destain with destaining solution.
8. Analyze fractions for co-purified substrate. Take 10 μl aliquot, add 10 μl of loading buffer and incubate at 37 °C for 3 h. Resolve samples (10 μl/lane) in Novex 4–20% TBE polyacrylamide gel using 1X TBE running buffer pre-chilled to 4 °C (200 V for 1 hour at 4 °C). Stain gel with 1X SYBR Gold dissolved in 1X TBE for 10 minutes. Wash gel with 1X TBE for 10 minutes. Scan gel for SYBR Gold fluorescence using Bio-Rad ChemiDoc XRS+.
9. Take peak fractions and supplement with 2 mM AMPPNP using quenching buffer. RNA loaded human MTR4-exosome complex can be used for subsequent cryo-EM grid preparation and analysis.

### 4.4 Notes

**Figure 5.**
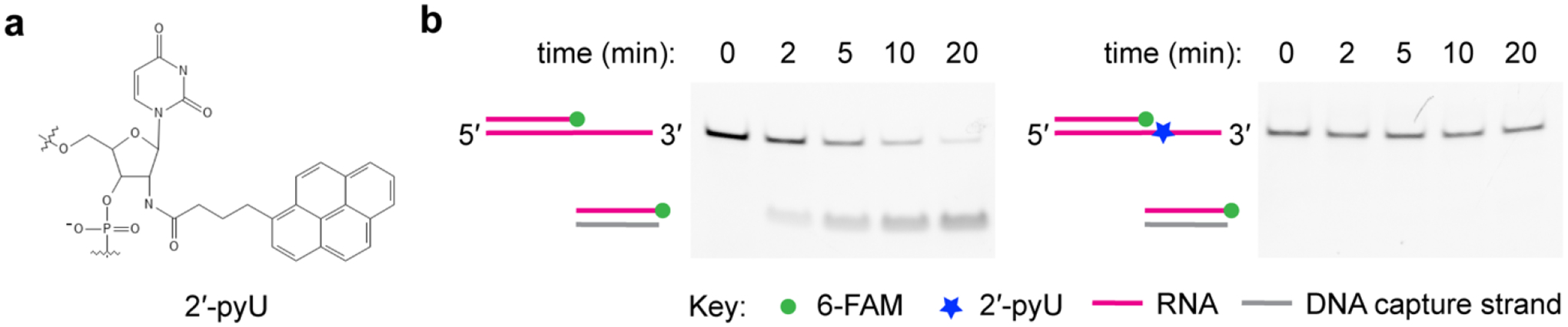
2′-pyU-modified substrate inhibits helicase activity. (a) Chemical structure of 2′-amino-butyryl-pyrene uridine modification. (b) Native PAGE showing time courses of NEXT unwinding reaction on a 3′ tailed RNA duplex substrate with (right panel) or without (left panel) 2′-pyU-modification.

- Bulky chemical moieties can be conjugated to a nucleotide in the translocation strand to block MTR4 tracking on the RNA substrate. For example, we found that incorporation of 2′-amino-butyryl-pyrene-modified uridine (2′pyU) in the translocation strand inhibits unwinding by NEXT (Fig. 5).

## Figures and Tables

**Table 1.** List of oligonucleotides for substrate preparation.

Oligo ID	Type	Sequence (5′ to 3′)	Label	Manufacturer	Assay
GS1	RNA	AGCACCGUAAAGACGC^a^	5′ 6-FAM	IDT	Gel shift (reporter strand)
GS2	RNA	GCGUCUUUACGGUGCUAAAAAAAAAAAAAAAAAAAA^a^	none	IDT	Gel shift (translocation strand)
MBHA1	RNA	AGUGCGCUGUAUCUUCAAGGCCACU^b^	5′ Iowa Black RQ 3′ Cy5	IDT	MBHA (molecular beacon strand)
MBHA2	RNA	AGUGGCCUUGAAGAUACAGCGCACUAAAAAAAAAAAAAAAAAAAA^b^	none	IDT	MBHA (translocation strand)
StrandA1	RNA	GCGTCTTTACGGTGCTCACCACACCACACCACACCACACCACACCACACCACACAAAAAAAA	none	Dharmacon	Gel shift (strand A)
StrandA2	RNA	GCGTCTTTACGGTGCTCACCACACCACACCACACCACACCACACCACACCACACAAAAAAAA	5′ Fluorescein	Dharmacon	Decay (strand A)
StrandA3	DNA/RNA	GCGTCTTTACGGTGCTCACCACACCACACCACACCACACCACACCACACCACACAAAAAAAA^c^	none	Dharmacon	Exosome loading (strand A)
StrandB1	DNA	GTGTGGTGTGGTGTGGTGTGGTGTGGTGTGGTGTGGT	none	Dharmacon	Decay (strand B)
StrandB2	DNA	GTGTGGTGTGGTGTGGTGTGGTGTGGTGTGGTGTGGT	5′ 6-FAM	IDT	Gel shift (strand B)
StrandB3	DNA	GTGTGGTGTGGTGTGGT	none	IDT	Exosome loading (strand B)
StrandC1	RNA	AGCACCGUAAAGACGC	none	Dharmacon	Gel shift/Decay/Exosome loading (strand C)
Capture1	DNA	GCGTCTTTACGGTGCT	none	IDT	Gel shift (capture strand)
Capture2	DNA	ACCACACCACACCACAC	none	IDT	Gel shift/Exosome loading (capture strand)

These oligonucleotide sequences are derived from ^a^Jia et al., 2012 or ^b^Belon and Frank, 2018.

c DNA sequences are underlined.
